# Effect of *Cosmos*, *Crotalaria, Foeniculum,* and *Canavalia* species, single-cropped or mixes, on the community of predatory arthropods

**DOI:** 10.1038/s41598-022-20188-6

**Published:** 2022-09-26

**Authors:** Adamastor Pereira Barros, Alessandra de Carvalho Silva, Antonio Carlos de Souza Abboud, Marcelo Perrone Ricalde, Julielson Oliveira Ataide

**Affiliations:** 1 Plant Science Department, Federal Rural University of Rio de Janeiro (UFRRJ), BR 465 km 47, Seropédica, RJ 23851-970 Brazil; 2 Embrapa Agrobiology, Brazilian Agricultural Research Corporation (Embrapa), BR 465, km 47, Seropédica, RJ 23891-000 Brazil; 3 Plant Science Department, Federal University of Espírito Santo (UFES), Guararema Alegre, Espírito Santo, 29500-000 Brazil

**Keywords:** Zoology, Entomology

## Abstract

Some plants can attract natural enemy by offering resources such as alternative food and refuge. However, studies need to be conducted before agricultural landscape diversification is implement. Our objective was to determine the best floristic compositions of cosmos (*Cosmos sulphureus*—Asteraceae), showy rattlepod (*Crotalaria spectabilis*—Fabaceae), fennel (*Foeniculum vulgare*—Apiaceae), and jack bean (*Canavalia ensiformis*—Fabaceae) to attract and maintain predatory arthropods, and know the potential of these treatments for future use in diversifying agricultural systems. The experimental design consisted in seven treatments of four species in single-crop, intercrops in three densities called mix1, mix2, and mix3, and the control (weeds). For the arthropod families classified as very frequent and constant, population dynamics in intercropping treatments was plotted according to the plant phenology. We conclude that all plants cultivated in single-cropping and intercropping treatments showed high predator richness and can potentially be used to diversify cultivated areas. Sulfur cosmos as a single crop and three mixes attracts higher numbers and greater family richness. Spider families—Oxyopidae, Araneidae and Thomisidae—and insects—Chrysopidae and Coccinellidae are more frequents. The dynamics of the predator populations varied according to the mixes treatment.

## Introduction

Agriculture has been advancing toward increasingly sustainable practices that aim to maximize the efficient use of natural resources. Several ecosystem services may be found in more diverse areas, especially those related to biocontrol of pests. Simplified agricultural systems, like monocultures, facilitate plant location by phytophages insects, making them more susceptible to pest attack^1^. Monocultures are also more vulnerable due to slow responses of trophic interactions caused by the reduced availability of refuge areas for biological control agents^2^.

Landscape complexity favors arthropod diversity, contributing to their stability, consequently allowing trophic interactions that naturally regulate crop pests^3^. In diversified crops, the refuge areas make trophic interactions more intense, minimize phytosanitary problems, and reduce interventions with pesticides^4^.

Among the strategies for attracting and maintaining natural enemies in crops, diversification through the addition of flowers and cover plants is an efficient and reproducible option to increase efficiency of biological pest control. Greater benefits occur when the plants added have multiple functions in the production system and contribute broadly to sustainability in addition to attracting natural enemies.

An example of multifunctionality is the cover plants commonly used as green manure, which can also contribute to weed and phytopathogen suppression as well as natural enemy attraction. Showy rattlepod (*Crotalaria spectabilis*—Fabaceae) is one of these plants; it controls nematodes, its pollen is used as food by predators like *Chrysoperla externa* (Neuroptera: Chrysopidae)^5^, and it harbors other natural enemies. In turn, jack bean (*Canavalia ensiformis*—Fabaceae) is a green manure that controls plant pathogens and offers a refuge for predatory arthropods^6^.

Plants of the Asteraceae and Apiaceae families are also attractive plants for parasitoids and predators^7^, and like Fabaceae, they have multiple functions in agricultural production areas. Sulfur cosmos (*Cosmos sulphureus*—Asteraceae) has the potential for floricultural production. Its flowers contain high concentrations of phenolic acids with herbicidal activity and mainly attract predators such as Coccinellidae and Syrphidae insects as well as Lycosidae and Linyphiidae spiders^8^. Fennel (*Foeniculum vulgare*—Apiaceae) is a known medicinal herb that is attractive to *Cycloneda sanguinea* (Coccinellidae), which is an outstanding biological control agent in various crops, such as vegetables and commodities. Other potentially attractive plants include Asteraceae, such as common sunflower (*Heliantus annuus*), Mexican sunflower (*Tithonia diversifolia* and *T. rotundifolia*), field chamomile (*Anthemis arvensis*), and cornflower (*Centaurea cyanus*), as well as Apiaceae, such as dill (*Anethum graveolens*), garden chervil (*Anthriscus cerefolium*), and coriander (*Coriandrum sativum*)^9–11^.

The diversification of agrosystems has not been a commonly adopted practice in Brazilian farmers, because of the lack of information about plants that attract natural enemies and their associated entomofauna. Although apparently simple, increasing the diversification of agricultural systems is quite complex, due to the formation of new interactions between organisms. According to Snyder^12^, the biggest challenge may be to extend knowledge about the effects of biodiversity on natural enemies and their enhancement through Conservation Biological Control (CBC) to species-rich tropical agroecosystems. Given this, we consider that the potential of plants to attract natural enemies needs to be carefully investigated before being tested with agricultural crops. Thus, the future compositions with agricultural crops to increase the functional diversity will be conditioned by beneficial entomofauna attracted by the previously tested plants.

Our objective was to determine the best floristic compositions of sulfur cosmos, showy rattlepod, fennel, and jack bean to attract and maintain predatory arthropods along with the potential of these treatments for future use in diversifying agricultural systems.

## Material and methods

The experiment was carried out under field conditions, in an experimental area at the Brazilian Agricultural Research Corporation, in Seropédica municipality (Latitude: 22° 44′ 29′′ S, Longitude: 43° 42′ 19′′ W), Rio de Janeiro State, Brazil.

**Figure 1:**
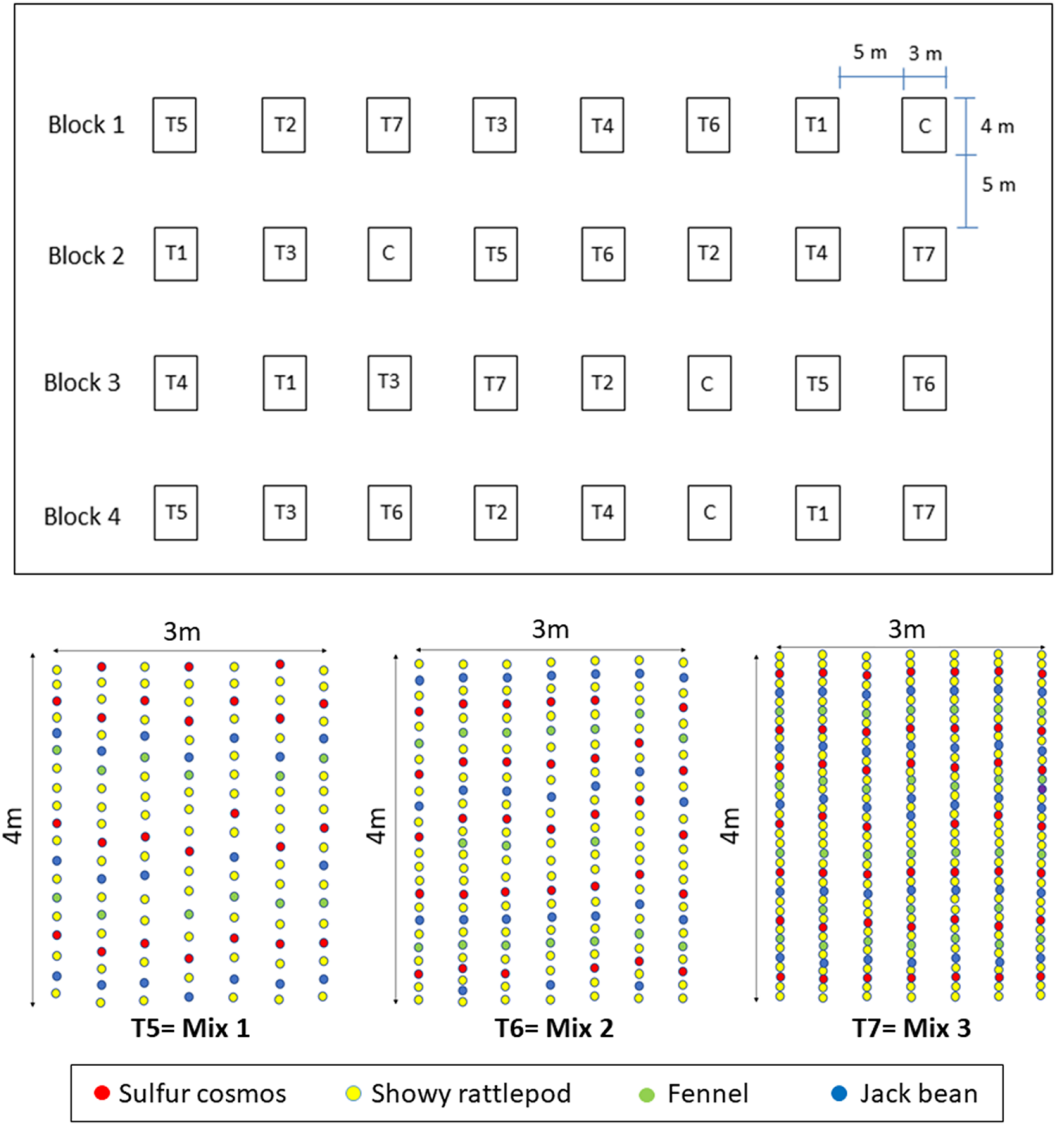
Experimental design. Seropédica, RJ, Brazil, May to November 2017. Treatments: T1: sulfur cosmos; T2: showy rattlepod; T3: fennel; T4: jack bean; T5: mix^a^ 1; T6: mix 2; T7: mix 3; C: control. ^a^Mix 1, 2, and 3 represent the intercropping of sulfur cosmos, showy rattlepod, fennel, and jack bean in three cultivation densities 10.8, 14.3, and 21.5 plants m^−2^, respectively.

The experimental design was planned with 3.0 × 4.0 m plots (12 m^2^), consisting of seven cultivation rows 0.50 m apart (Fig. 1). The size of the plots was chosen to take into account the sampling effort and the maintenance of the area. To avoid interference between the plots, they were 5 m apart, and this area was kept without plants. The limits of the experimental area included: a road, a strip of *Gliricidia sepium* (Fabaceae), a fallow area, and experimental cultivation of rice and soybean, with distances of 15 m each. A forest fragment was located more than 200 m from the plots. The areas between the surrounding vegetation and the experimental plots also were kept without plants to reduce interference in our study. A randomized complete block design was adopted to dilute the spatial auto correlation effect and control the effects due to the slope of the terrain, patch of soil, and arboreal/shrub vegetation that could compete with the attractiveness of the evaluated plants. Greater distances between the plots of 20, 50, or 100 m would accommodate different soil textures, air currents, and an unwanted edge effect such as other crops and forest fragments.

Four blocks, seven treatments, and one control were adopted. The treatments consisted of four plant species (sulfur cosmos, showy rattlepod, fennel, and jack beans) panted in single-croppings and three mixes of the four plants (in three different densities). The densities used in single-croppings were based on Hogg et al.^13^ and Ramalho et al.^14^. The three treatments called mixes contained densities equal to 1/4, 1/3, and 1/2 of the density used in the monoculture (Fig. 1, Table 1). Weeds, naturally occurring and uncontrolled, were used as the control, representing a cover which also offers resources for natural enemies^15^ without human intervention. To calculate the plant density of the plots with weeds, a hollow wooden square with 1 m^2^ was cast over the four plots with this coverage. The plants were counted, and the density was determined through the average of the four replications (Table 1).

**Table 1:** Plants density in the assessed treatments. Seropédica, RJ, Brazil, May to November 2017.

Treatments	Plant density (number of plants m^−2^)				
	Sulfur cosmos^1^	Showy rattlepod^1^	Fennel^1^	Jack bean^1^	Total
Single-cropping 1	8	–	–	–	8
Single-cropping 2	–	25	–	–	25
Single-cropping 3	–	–	4	–	4
Single-cropping 4	–	–	–	6	6
Mix 1 (1/4)	2	6.3	1	1.5	10.8
Mix 2 (1/3)	2.7	8.3	1.3	2	14.3
Mix 3 (1/2)	4	12.5	2	3	21.5
Control^2^					87

^1^sulfur cosmos = *Cosmos sulphureus* (Asteraceae)*;* showy rattlepod = *Crotalaria spectabilis* (Fabaceae); fennel = *Foeniculum vulgare* (Apiaceae); jack bean = *Canavalia ensiformis* (Fabaceae). ^2^Weeds (12 botanical species m−2 on average).

Fennel seedlings were sown in trays up to 50 days before transplanting; sulfur cosmos and showy rattlepod were sown in the field on the same date as fennel transplanting: 15/05/2017. Jack beans were sown 15 days after the others due to its faster and vigorous growth. The plants were kept in the field from May to November 2017.

The arthropod samplings were performed every 15 days, between the 4 July and 3 November 2017, totaling nine samplings (04/07, 20/07, 03/08, 19/08, 30/08, 18/09, 2/10, 18/10, 3/11/2017). Predatory insects and spiders were captured using an adapted motorized vacuum (STIHL BG86C) with a voile fabric bag attached to the vacuum tube inlet (see Supplementary Fig. S2 online). Vacuuming was done over and between the plants for 30 s^16^, in a 1 m^2^ area at the center of plots. The predators were identified to the family level and classified into morphotypes.

As aphids had a high incidence of fennel during the flowering period of this plant, they were also sampled with the same motorized vacuum model used to sample predators. After collection, the aphids were taken to the laboratory at Embrapa Agrobiology, for counting winged and non-winged adults, with the aid of a 40 × LED binocular stereoscope and a manual numerical counter. A sample of the aphids was sent to the ‘Marcos Enrietti Diagnostic Center’ at the Federal University of Paraná (UFPR) for identification by Dr. Regina Celia Zonta de Carvalho.

To determine the flowering intensity in the mixes (Fig. 3), the floral units of the plants that compose them were quantified weekly. For sulfur cosmos, showy rattlepod, and jack bean, the flower buds were considered in the pre-anthesis stage. For fennel, the umbel in anthesis was considered as a floral unit. All floral units at 1 m^2^ inside in the mixed plots were counted, and based on the known plant density, the flowering intensity (percentage) in each mix treatment was calculated.

The plant material of the treatments was not classified for this study and deposited in institutional herbaria, because these are known botanical species. The arthropods captured in cultivated plants and weeds that make up the control were registered and authorized in the Biodiversity Information System (SISBIO) under number 73052–1. The use of plants was in accordance with national legislation. All plants and insects were registered in the National Management System for the Genetic Heritage and Associated Traditional Knowledge (SisGen), which is required to authorize research in Brazil.

### Analyses

The frequency, constancy, and richness of the arthropod families collected in the treatments were calculated based on Silveira Neto et al.^17^. The population dynamic charts and ANOVA were done only when the predatory arthropod family frequency was equal to or higher than the upper limit of the confidence interval (CI), at 5% probability. The confidence interval was calculated from the equation CI = m ± t*s(m), where: m = mean frequency estimate; t = 2.04 for α = 5% with 31 degrees of freedom; s(m) = standard error of the mean. These families were called “very frequent.” The families were considered “constant” when found in 50% or more of the samples.

To assess the faunistic parameters of the arthropod predator communities, Rényi diversity profiles were performed. The profile values for alpha = 0, 1, 2, and infinity indicating the species richness, Shannon diversity index, logarithm of the reciprocal Simpson diversity index, and Berger-Parker diversity index, respectively. If the profile for one sample was consistently higher than the profile for another sample, the sample with the higher profile was considered more diverse. Intersecting curves for two communities indicate that they cannot be ranked.

To assess the relationship between plant densities in mixes 1, 2, and 3 as well as the predators, only the predator families belonging to the categories ‘very frequent’ and ‘constant’ (Araneidae, Coccinellidae, Chrysopidae, Oxyopidae, and Thomisidae) in the three mixes were used. The population dynamics of these predators and fennel aphids were plotted with the flowering time of the plants in the mixes (Fig. 3). To compare on a graphic scale the population fluctuation of predators with the population of aphids captured on fennel, the mean values of aphids were transformed (√x)/5). This transformation was necessary because the population of aphids is much higher than that of natural enemies (means of aphids had three decimal places and predators only two).

To determine the interference of the plant density in the mixes on the population of the main predators, Pearson’s correlation was performed, using the PAST statistical program: five families of predators (Araneidae, Coccinellidae, Chrysopidae, Oxyopidae and Thomisidae) × number of plants.m-2 (density in mixes 1, 2, and 3) (Fig. 4).

## Results

In the four studied plants and the control, 2,890 predatory arthropods were collected, including in the single-cropping and intercropping systems, in nine samplings that occurred in the period July to November 2017. We did not identify edge effects in our plots or blocks, although our plots occupied an area larger than 50 m.

The single-cropping of sulfur cosmos and the three mixes contained the highest number of predators among the treatments and were superior to the control (weeds) (p < 0.05). Predator family richness observed on sulfur cosmos and the mixes surpassed the values found in the other treatments and were the same as the control (p < 0.05). The number of constant families captured on the mixes were same as the control and both were statistically higher (p < 0.05) than on other treatments in single-crops. The number of frequent families did not differ statistically between the treatments and the control. The density increase in the mixes did not affect the faunistic parameters tested (Table 2).

**Table 2:** Faunistic parameters of predatory arthropods captured in plants under single-cropping and mixes. Seropédica, RJ, Brazil, July to November 2017.

Faunistic parameters	Treatments							Control (weeds)	CV% ^b^
	sulfur cosmos	showy rattlepod	fennel	jack bean	mix 1^a^	mix 2^a^	mix 3^a^		
Mean no. of predators^c^	108.2a	36.0d	74.0c	30.5d	111.0a	129.7a	144.7a	85.2b	24.1
Mean no. of richness of families^c^	18.0a	9.25b	10.0b	11.5b	13.75a	14.0a	15.75a	15.75a	14.12
Mean no. of constant families^c.d^	2.75b	2.00b	2.50b	1.50b	4.0a	4.25a	4.0a	3.25a	40.82
Mean no. of frequent families^e^	5	5	4	6.25	5.50	5.25	5.0	6.25	24.32

^a^Mix 1, 2, and 3 represent the intercropping of sulfur cosmos, showy rattlepod, fennel, and jack bean in three cultivation densities 10.8, 14.3, and 21.5 plants m^−2^, respectively; ^b^CV = Coefficient of variation; ^c^Means followed by the same letter on the line do not statistically differ by the Scott-Knott test (p < 0.05); ^d^Presence higher than 50% in samples; ^e^Frequency higher than the upper confidence interval (p < 0.05), which indicates that do not differ from each other by the F test (p < 0.05).

### Community of predatory arthropods associated with different floristic compositions

The highest predator richness found in this study belongs to the order Araneae (class Arachnida), which was equivalent to 36.6% of the family found in the assessed area. The other predators were insects of five orders: Coleoptera (16.7%), Hemiptera (16.7%), Hymenoptera (16.7%), Diptera (10%), and Neuroptera (3.3%). From the 31 families found, nine occurred in all treatments: Araneidae, Eutichuridae, Oxyopidae, Thomisidae, Salticidae, Carabidae, Coccinellidae, Formicidae, and Chrysopidae. Some of these families were more prevalent in certain treatments, while their occurrence was less relevant in others.

**Figure 2:**
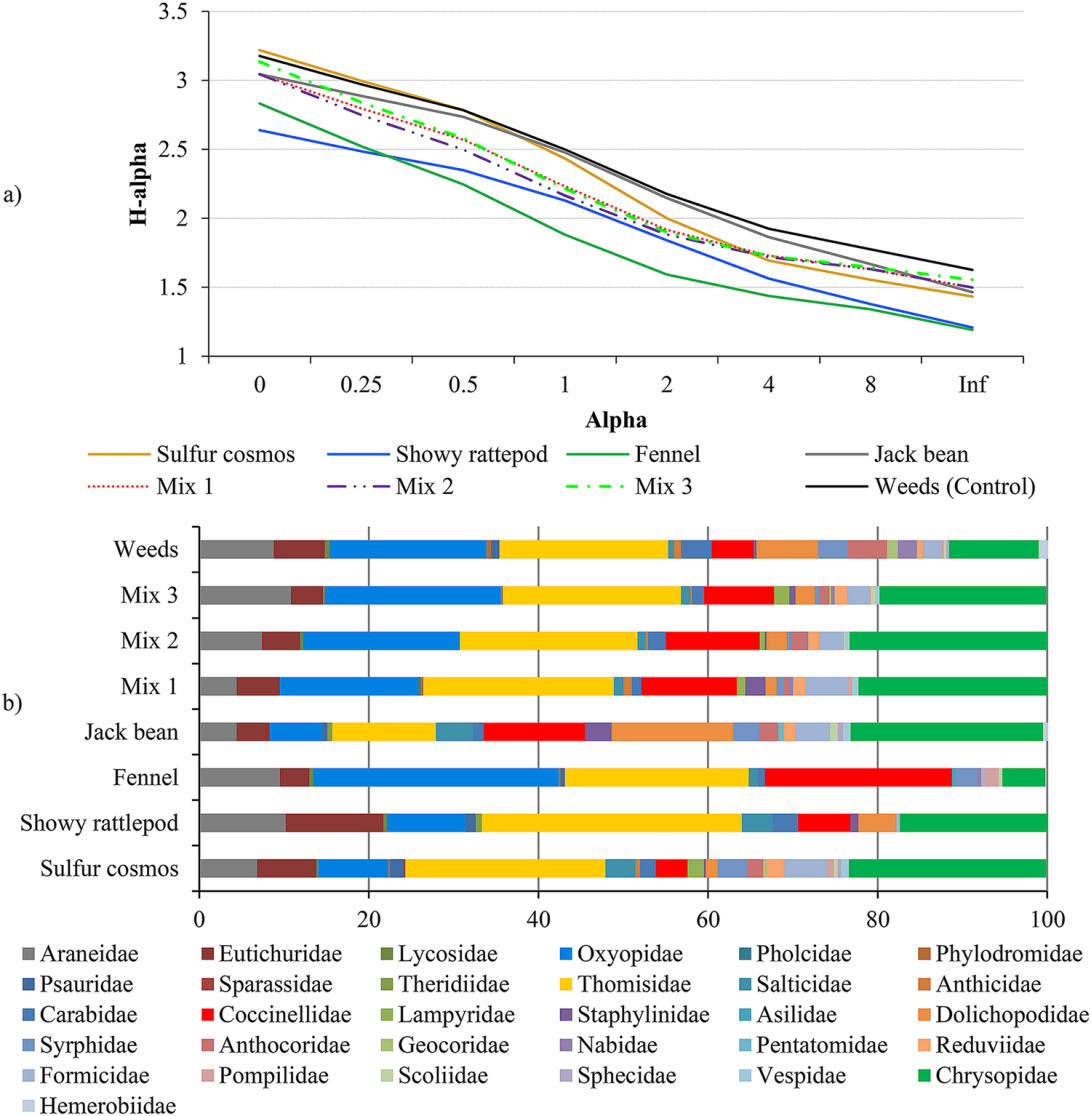
*Rényi diversity profiles* (**a**) and mean frequency of predatory arthropods (**b**) captured in single-cropping and intercropped systems (mixes)^a^, and weeds (control). Seropédica, RJ, Brazil, July to November 2017. ^a^Mix1, 2, and 3 represent the intercropping of sulfur cosmos (*Cosmos sulphureus*), showy rattlepod (*Crotalaria spectabilis*), fennel (*Foeniculum vulgare*), and jack bean (*Canavalia ensiformis*) in three cultivation densities 10.8, 14.3, and 21.5 plants m^−2^, respectively.

The Rényi profile was traced to analyze the characteristics of the predatory arthropod community in our study (Fig. 2a). The seven treatments and the control did not have horizontal profiles, as their curves decline from left to right. This indicates that the families of insects and spiders were not equally distributed in the treatments (there is no equitability) and that some were dominant. In general, the dominant families were the spiders Oxyopidae, Thomisidae, and Araneidae as well as the insects Chrysopidae, Coccinellidae, and Dolichopodidae (Fig. 2b).

However, the percentage varied that each dominant family occurs in the treatments, as observed in infinite alpha (Fig. 2a). Profiles with higher infinite alpha had a lower percentage of the dominant species, that is, the species present in the treatment were more equitable. Thus, in mix 3 and the control (weeds), located at the highest infinite alpha, the percentages of predators from the families Oxyopidae, Thomisidae, and Chrysopidae were 19.06, 19.65, 11.44% and 21.11, 20.93, 19.72%, respectively. However, in plants with lower infinite alpha (Fig. 2a), the percentage of dominant families was high: fennel [Oxyopidae (30.41%), Thomisidae (22.30%), and Coccinellidae (21.36%)], showy rattlesnake [Thomisidae (29.86%) and Chrysopidae (17.36%)] (Fig. 2b). In the other treatments, the frequency of predatory arthropods was intermediate (Fig. 2a). All these families were considered constant in these treatments, as they occurred in more than 50% of the samples performed.

According to Rényi profile, the greatest richness of families (observed at alpha = 0) was found in the sulfur cosmos (25 families), followed by weeds (24) and mix 3 (23). The other two mixes (mix 1 and 2) had a richness equal to that of jack beans (21), which in turn were superior to that of fennel (17). The lowest richness of predators was observed for showy rattlepod (14) (Fig. 2 a,b).

### Population dynamics of predatory arthropods in plant mixes

**Figure 3:**
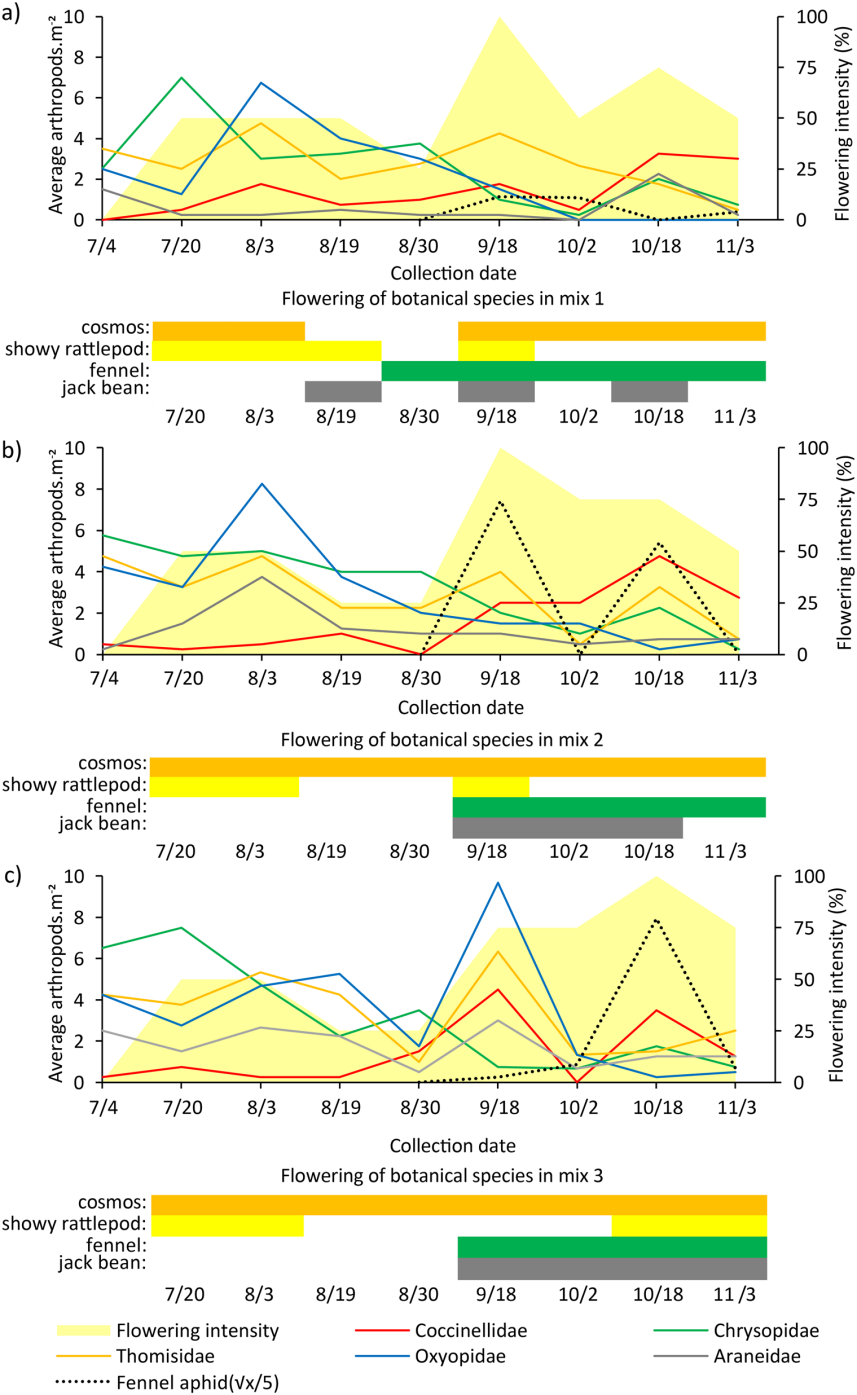
Population dynamics of very frequent and constant predatory arthropods and the aphid *Hyadaphis* cf. *foeniculi*^a^ (Hemiptera: Aphididae) as a function of the flowering intensity of the intercropped plants^b^: mix 1 (**a**), mix 2 (**b**), and mix 3 (**c**). Seropédica, RJ, Brazil, July to November 2017. ^a^Data transformed to (√x / 5); ^b^ Mix 1, 2, and 3 represent the intercropping of sulfur cosmos, showy rattlepod, fennel, and jack bean in three cultivation densities 10.8, 14.3, and 21.5 plants m^−2^, respectively.

The combination of four plant species in the mixes resulted in a constant supply of flowers in the three densities studied. As expected, the flowering varied according to the mean density of the constituent species (11, 14, and 22 plants m^−2^). Flowering intensification was verified in the second half of the study cycle (from August 30). Flowering peaks correspond to the presence of flowers in all four plant species evaluated (Fig. 3).

The population dynamics of predators in the mixes was modified with increased plant density in these three treatments. During most sampling periods, Coccinellidae, Chrysopidae, Oxyopidae, Thomisidae, and Araneidae predators coexisted in the three mixes. Although, the number of arthropods varied with peaks occurring at different times during the four months (Fig. 3).

**Figure 4:**
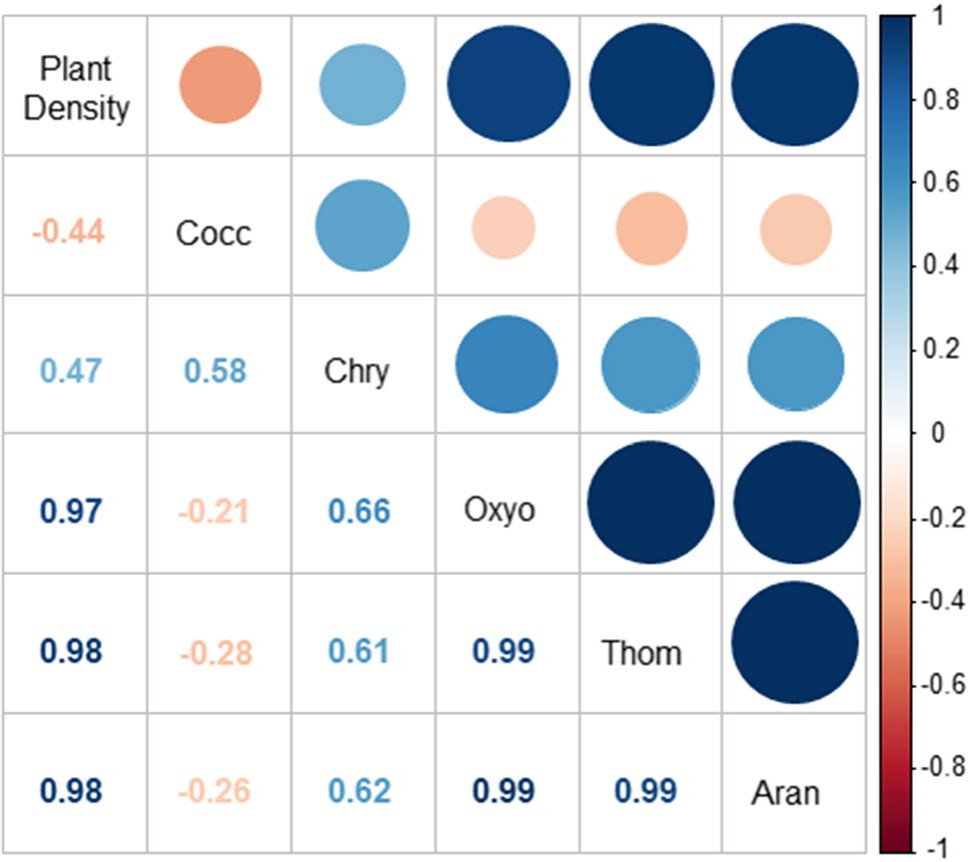
Pearson correlation matrix of the most important predatory arthropod families in three plant densities on intercropping systems. Seropédica, RJ, Brazil, July to November 2017. PD—Plant density; Cocc—Coccinellidae; Chry—Chrysopidae; Oxyo—Oxyopidae; Thom—Thomisidae; Aran—Araneidae.

The population of the Araneidae weaver spiders increased as the plant density increased in the mixes (Fig. 3). In mixes 1 and 2 (Fig. 3a,b), Chrysopidae insects as well as Oxyopidae and Thomisidae spiders occurred mostly in the first half of the experiment, when only sulfur cosmos and showy rattlepod were flowering, and reduced in the last three sampling dates, when the other two plant species were also in bloom. After the second half of October in mix 1, and in September and mixes 2 and 3, other predators such as Coccinellidae became predominant in the refuges with these configurations. There was no significant correlation between coccinellids and plant density in the mixes (r = −0.44) (Fig. 4).

The increase of the flowering of fennel in the mixes coincided with the recording of aphids *Hyadaphis foeniculi* (Hemiptera: Aphididae). This pest occurred in greater numbers in mixes 2 and 3. Mix 2 suffered two aphid population peaks and mix 3 had a single population peak, both in October (Fig. 3b,c).

Although they occurred at the same time, Coccinellidae and Chrysopidae had a low correlation with the three spider families (r = 0.66; 0.61, and 0.62 for Oxyopidae, Thomisidae, and Araneidae, respectively) and with the plant density (r = 0.47). In contrast, these spiders had a strong positive correlation between each other (r = 0.99) and with increasing plant density in the mixes (r = 0.97 or 0.98) (Fig. 4).

## Discussion

### Potential for attracting and maintaining predators

In our study, the diversity of predators in single- and intercropping systems is clear. Although the different treatments hosted species in common, the importance that each species had in the treatment was different (Fig. 2), which was expected. According to Sivinski et al.^18^, each plant has the potential to attract a specific or broader group of natural enemies, according to its floristic, morphological, and biochemical characteristics. For Aldini et al.^8^, flowers can differ in their morphology, nectar, and pollen composition as well as emitted volatile compounds.

The predators attracted to the single-cropping of sulfur cosmos indicates that this plant could be a good option for diversification of agricultural crops due to the number of individuals and richness of families associated with it, comparable to the mixes. It was the only individual plant species studied that performed this well. The other plant species presented statistically lower contributions (p < 0.05) in the single-croppings than in the mixes, for most of the fauna parameters evaluated (Table 2). In the mixes, the sulfur cosmos also exhibited good adaptation to the increase in plant density, with continuous flowering in mix 2 and mix 3 (Fig. 3), which makes it a great supplier of floral resources such as nectar and pollen. Aldini et al.^8^ also observed the great attractiveness of the cosmos for natural enemies and stated that spiders can benefit from the refuge conditions provided by the dense and branched plant architecture. Another characteristic of the cosmos associated with natural enemies is its capitulum-like inflorescence and ligulate corolla, whose mature pollen is available for insects to forage^19,20^.

Although in our study the sulfur cosmos was associated with spiders Araneidae, Eutichuridae, Oxyopidae, and Thomisidae, in addition to insects of the Chrysopidae family, these are not always the natural enemies associated with this plant. In a study carried out with sulfur cosmos in California, predators were infrequent and diverse, and the parasitoids were most associated with this plant^13^. Aldini et al.^8^ found thirty families of predators attracted to this plant in a study conducted in Indonesia, where Lycosidae, Linyphiidae, Syrphidae, and Coccinellidae stood out; the families Thomisidae and Chrysopidae, which were the most frequent in our study, were not found by the authors.

As for fennel, research conducted in Brazil and the United States has shown that its flowers are attractive to predators from several families, including Coccinellidae, Sphecidae, and Vespidae^21–23^,of these families, only the Coccinellidae appears in all three studies. In our study, ladybugs are as frequent in fennel as the spiders Oxyopidae and Thomisidae. The wasps Sphecidae and Vespidae may not have been captured in our study because they fly fast and tend to flee when threatened, and the noise of the motorized vacuum may have driven them away.

Eggs, larvae, pupae, and adults of coccinellids were found in fennel, before and during flowering, demonstrating the ability of this plant to provide food and breeding sites. However, the increase of these predators coincides with the flowering of fennel and with the recording of the aphid *H. foeniculi* in the inflorescences of this plant, both in the mixes (Fig. 3) and in the single-cropping. In the single-cropping of fennel, the population peaks of the aphid *H. foeniculi* were observed at the same time in the month of October as in the mixes. However, the aphids peaks in the single-cropping were higher (average of 583 and 586.75 aphids/m^2^ in the collections on 02/10 and 18/10) than in the mixes [average 275 and 147 aphids/m^2^ in mix 2 (18/9 and 18/10) and mix 3 (314.5 aphids/m^2^ in 18/10), respectively]. These data show that the food resources provided to aphidophagous predators are offered directly (through pollen and shelter) and indirectly (through prey). The relationship between ladybugs, fennel flowering, and occurrence of aphids was also observed by Lixa et al. ^22^ and Balzan et al. ^24^.

In relation to Fabaceae, showy rattlepod and jack bean in single-crops had different patterns of attractiveness to predators. The first had a lower richness of families (14) and this community was predominantly formed by spiders. The jack bean had a greater richness of predators (21) and a lower frequency of spiders than the first but was favorable for insects such as ladybugs, Dolichopodidae flies, and lacewings (Fig. 2). The flowering of these two Fabaceae was delayed in the mixes, compared to the single-cropping. This change in the beginning of flowering occurred as a consequence of the shading caused by the cosmos in mixes 1, 2, and 3.

The greater richness of predators in the single-cropping of jack bean probably occurred due to the greater production of phytomass and soil cover, producing more refuge areas and allowing the coexistence of several species in the plant. The more erect posture and the flowers of showy rattlepod facilitated the construction of webs and the ambushes by the spiders. Carvalho et al.^25^ evaluated the intercropping of jack beans with citrus and observed an increase in natural enemies (coccinellids, lacewings, spiders, and parasitoids) and these were responsible for controlling Orthezia and reducing phytophagous mites. Lisboa et al.^26^ evaluated the presence of natural enemies in *C. spectabilis* between the rows of a passion fruit orchard and found a predominance of spiders and, other predators, such as coccinellids, lacewings, and carabids, in smaller numbers.

Green lacewings and coccinellids are active predators of mealybugs, aphids, eggs, and larvae of lepidopterans, whiteflies, and mites^27,28^. They contribute to the regulation of insect pests, although they also use pollen and nectar to supplement their diet^29,30^. Spiders are generally more generalists, especially those that use webs, and some species that are hunters may have a diet restricted to flies and small insects^31,32^. Dolichopodids feed on small, soft-bodied arthropods, preferably larvae of other dipterans, some hemipterans, mites, and thrips^33^.

### Superiority of mixes

Our results indicate that the greater plant diversity achieved in mixed systems favors predators, especially generalists, including spiders. This was observed both in the natural systems with weed cover (control) and in the systems created in our study, with plant mixes.

The superiority of mixed treatments to most monocultures, with regard to the presence of beneficial arthropods (Table 2), can be explained by the biotic and abiotic complementarity created by plants with distinct botanical characteristics. In refuges with greater plant diversity, more complex structures such as branches, leaves, flowers, and leaf litter occur, which favor refuges. According to Khaliq et al.^34^, different combinations of plants are also capable of altering the microclimate. According to the authors, in a monoculture landscape, refuges function like trees in a pasture. The microclimate of the refuges offers greater thermal comfort, sun and wind protection, higher relative humidity through plant evapotranspiration, and more abundant floral resources and prey. In response to these factors, predators can express their full biotic potential increasing their diversity, constancy, and frequency.

The prey menu available to predators in diverse habitats can be made up of phytophagous, pollinators, detritivores, and other predators^35^. This condition can create three situations: a) a complementary action of predators, increasing the suppression effect of pest arthropod populations,b) a negative interaction, such as intraguild predation, in which a predator becomes the prey of another predator; c) competition between predators for the same resources, resulting in the extinction of one of them at that location. However, refuges containing several plants can limit encounters between predators, which brings intraguild predation to equilibrium levels, allowing the coexistence of species^36^ and contributing to the maintenance of predator diversity.

Even on a small scale and in landscapes dominated by monocultures, landscape diversification can help reduce pests, as shown by some studies. Perennial ryegrass (*Lolium perenne*—Poaceae) intercropped with white mustard (*Sinapis alba*—Brassicaceae) and white clover (*Trifolium repens*—Fabaceae) between the rows of pears increased the diversity, predators, and phytophagous indices, overcoming the effect of cover by spontaneous plants^37^. Consortia with Fabaceae, such as *Crotalaria juncea* and *Mucuna pruriens*, attracted natural enemies and reduced pest levels in zucchini (*Curcubita pepo*—Curcubitaceae) cultivation^38^.

Regarding the use of mixes in the diversification of agricultural crops, Campbell et al.^39^ showed that a multifunctional mix of flowers in apple orchards helped to increase the fauna of beneficial insects in the culture. Staton et al.^40^ also evaluated flower mix in apple orchards managed with and without cutting and observed that plots maintained with flowering decreased the aphid attacks and increased the pollination of apple trees.

The presence of Thomisidae and Chrysopidae in 77.8 to 83.6% of the samples in the mixes we studied showed that the environment provided resources for natural enemies for a long time. The concomitant presence of the two predator families in the mixes, from June to October (Fig. 3), could also indicate that predators from these two families did not compete for the same resources or that the mixes provided sufficient resources for both. In addition to Thomisidae and Chrysopidae, other predators such as Oxyopidae, Araneidae, and Coccinellidae were constant in the mixes in more than 50% of the collections during the study period, accompanying the supply of flowers in these treatments.

Quality and diversity are decisive factors in the establishment of floristic compositions for the attractiveness and stability of natural enemy communities on a constant basis. The importance of diversity explains why the three mixtures did not show statistical differences for faunal parameters such as average number of predators, richness, and constancy of families, but they did for most plants in single-cropping (Table 2). The quality of plants that offer mixed diets that include prey and pollen supplementation increases the development, survival, fecundity, and longevity of different predators, such as *Orius* spp. (Anthocoridae), *Geocoris punctipes* (Geocoridae), and other families such as Lygaeidae, Pentatomidae, Nabidae, and Reduviidae^41^, and Chrysopidae, Coccinellidae^42,43^, as well as spiders^44^.

### Spider community

Under the conditions studied, we found a high frequency of spiders, both in single-cropping and in intercropping. Spiders often accounted for more than 50% of all predators encountered (Fig. 2b). This demonstrates that all plants studied have the necessary complexity for this association with Arachnids, especially those belonging to the Araneidae, Oxyopidae, and Thomisidae families. According to Symondson et al.^31^, more complex landscapes are inhabited by omnivorous generalist predators, mainly spiders. However, according to Sunderland and Samu^45^, areas with greater vegetation cover, such as in intercropping, favor members of the Oxyopidae, Thomisidae, Araneidae, and Salticidae families, as they provide better refuge and protection than single-cropping. As top predators, spiders also require food quality, in addition to the structural composition and complexity of the plant habitat.

Spiders have different predation behaviors^32^. Araneids (Araneidae) are aerial orb weavers, which take advantage of stems, sheaths, leaves, and flowers to make their webs and intercept flying Lepidoptera, Coleoptera, and Hymenoptera^46^. In our study, these spiders were more frequent on showy rattlepod and fennel, in addition to the mixes. As already mentioned, in the mixes, a higher occurrence of Araneidae was observed with greater density of plants (r = 0.98) (Figs. 2 and 4), which may have facilitated the establishment of spiders to capture prey.

Oxyopidae spiders are diurnal hunters, found in higher plant strata. They can prey on Hymenoptera (Apidae), Diptera, Lepidoptera, and Hemiptera (Miridae) in crops such as cotton, alfalfa, and soybean, as well as others of agricultural importance^47,48^. According to Winkler et al.^49^, the floral morphology of fennel in the form of an umbrella, with exposed nectaries, facilitates the accessibility of oxyopids to nectar. This energy supply is essential for successful leaps and ambushes to capture floral visitors.

Tomysids inhabit leaves and flowers, places used for ambushes against their prey, such as Hymenoptera, which can be agricultural pests^46^. According to Balzan et al.^24^, fennel is attractive to these spiders because the yellow flowers are used as ultraviolet (UV) patterns to attract and confuse floral visitors^50^. The high constancy and frequency associated with the yellow flowered plants in our study (sulfur cosmos, showy rattlepod, and fennel) suggest that this is an essential feature of predator attractiveness, since this effect is not observed in jack beans, which has purple-violet flowers.

In short, generalist predators are important to maintain insect pest population levels below the economic damage level in agricultural systems, which indicates that the studied plants have this aptitude, both when cultivated alone and when mixed with each other, at different densities. These annual plants provided other benefits in addition to attractiveness to predators, which makes this study different from the others. The multifunctionality of plants is related to the improvement of soil nutrition through nitrogen fixation by showy rattlepod and jack bean, to the aesthetic value of the flowers of the sulfur cosmos and the pharmacological value of fennel. These characteristics, in addition to the good soil cover provided by the mixes and knowledge about their maintenance in the field, make them superior to the control (weeds).

Agricultural habitats are often disturbed by cultural practices, which negatively affect the behavior of natural enemies, especially spiders. When refuge sites are maintained to host natural enemies, they resemble natural refuges, as they function as ecological infrastructure capable of providing resources, such as prey and alternative hosts; supplementary food; mating sites; and shelter from adverse agricultural practices including applications of insecticides, fungicides, or herbicides^51^. Therefore, refuges with flowering plants and with more complex architecture provided by the proposed intercropping work as natural breeding sites and origin of predators for dispersion and settlement in the cultivation system, in search of prey.

## Conclusion

Sulfur cosmos, showy rattlepod, fennel, and jack bean cultivated in single- and intercropped cultivation promoted predator richness and could be used in the diversification of agricultural crops. Among the monocultures, the sulfur cosmos was superior for having a higher frequency and richness of predators. Mixes grown in the three densities are more suitable for crop diversification than most monocultures. The treatments tested attracted and maintained generalist predators, mainly spiders, Chrysopidae, and Coccinellidae.

## Supplementary Information

Supplementary Information 1.

Supplementary Information 2.

Supplementary Information 3.

## Data Availability

All data generated or analyzed during this study are included in this published article (and its Supplementary Information files).
