# Supplementary figures and images for: Effect of Cosmos, Crotalaria, Foeniculum, and Canavalia species, single-cropped or mixes, on the community of predatory arthropods
Source: Sci Rep. 2022 Sep 26;12:16013. doi: 10.1038/s41598-022-20188-6 (PMC9512904; doi:10.1038/s41598-022-20188-6)

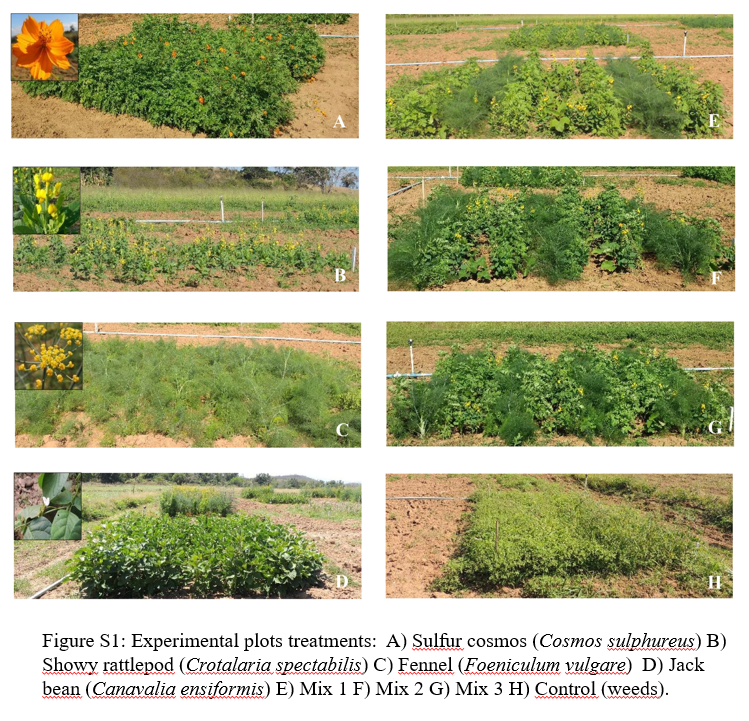

Supplement: Supplementary file 1 — Supplementary Information 1. [file 41598_2022_20188_MOESM1_ESM.tif]

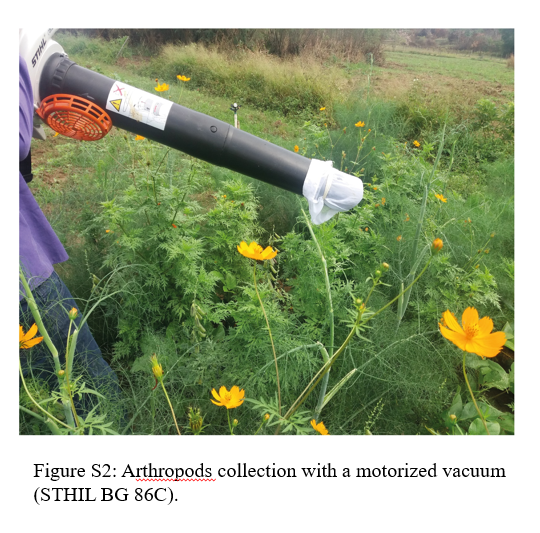

Supplement: Supplementary file 2 — Supplementary Information 2. [file 41598_2022_20188_MOESM2_ESM.tif]
